# A Network-Based Gene Expression Signature Informs Prognosis and Treatment for Colorectal Cancer Patients

**DOI:** 10.1371/journal.pone.0041292

**Published:** 2012-07-23

**Authors:** Mingguang Shi, R. Daniel Beauchamp, Bing Zhang

**Affiliations:** 1 Department of Biomedical Informatics, Vanderbilt University School of Medicine, Nashville, Tennessee, United States of America; 2 Department of Cancer Biology, Vanderbilt University School of Medicine, Nashville, Tennessee, United States of America; 3 Department of Surgery, Vanderbilt University School of Medicine, Nashville, Tennessee, United States of America; 4 Department of Cell and Developmental Biology, Vanderbilt University School of Medicine, Nashville, Tennessee, United States of America; 5 Vanderbilt-Ingram Cancer Center, Vanderbilt University School of Medicine, Nashville, Tennessee, United States of America; The George Washington University, United States of America

## Abstract

**Background:**

Several studies have reported gene expression signatures that predict recurrence risk in stage II and III colorectal cancer (CRC) patients with minimal gene membership overlap and undefined biological relevance. The goal of this study was to investigate biological themes underlying these signatures, to infer genes of potential mechanistic importance to the CRC recurrence phenotype and to test whether accurate prognostic models can be developed using mechanistically important genes.

**Methods and Findings:**

We investigated eight published CRC gene expression signatures and found no functional convergence in Gene Ontology enrichment analysis. Using a random walk-based approach, we integrated these signatures and publicly available somatic mutation data on a protein-protein interaction network and inferred 487 genes that were plausible candidate molecular underpinnings for the CRC recurrence phenotype. We named the list of 487 genes a NEM signature because it integrated information from Network, Expression, and Mutation. The signature showed significant enrichment in four biological processes closely related to cancer pathophysiology and provided good coverage of known oncogenes, tumor suppressors, and CRC-related signaling pathways. A NEM signature-based Survival Support Vector Machine prognostic model was trained using a microarray gene expression dataset and tested on an independent dataset. The model-based scores showed a 75.7% concordance with the real survival data and separated patients into two groups with significantly different relapse-free survival (*p* = 0.002). Similar results were obtained with reversed training and testing datasets (*p* = 0.007). Furthermore, adjuvant chemotherapy was significantly associated with prolonged survival of the high-risk patients (*p* = 0.006), but not beneficial to the low-risk patients (*p* = 0.491).

**Conclusions:**

The NEM signature not only reflects CRC biology but also informs patient prognosis and treatment response. Thus, the network-based data integration method provides a convergence between biological relevance and clinical usefulness in gene signature development.

## Introduction

Colorectal cancer (CRC) is the third leading cause of global cancer mortality [1]. According to stages defined by the American Joint Committee on Cancer (AJCC), 5-year survival rates are 93.2% for stage I, 82.5% for stage II, 59.5% for stage III, and 8.1% for stage IV CRC patients [2]. Adjuvant chemotherapy (CTX) for stage III CRC patients has demonstrated survival benefit; however, 42–44% of patients treated by surgery alone will not recur in 5 years [3]. On the other hand, although individual clinical trials have often failed to demonstrate the benefits of adjuvant CTX for stage II patients, approximately 20% of stage II patients will recur within 5 years. Hence, it is crucial to develop an accurate method for stratifying stage II and III CRC patients by risk of recurrence so that adjuvant CTX can be administered to high-risk patients, while low-risk patients can forgo these toxic treatments to avoid potential harm as well as the financial burden.

Based on the direct comparison of microarray data from highly aggressive and less aggressive CRC tumors, several studies have reported gene expression signatures that predict recurrence risk in stage II and III CRC patients [4], [5], [6], [7], [8], [9], with minimal overlap of their gene lists [10]. Lack of concordance is a common observation in gene expression signature studies [11], raising questions about their clinical implications [12]. However, prognostic models based on several CRC gene expression signatures have been validated in independent patient cohorts [6], [7], [8]. Moreover, an early study in breast cancer has demonstrated that apparently distinct signatures can show a significant agreement in outcome prediction [13]. It has been suggested that different signatures may share common biological themes that are not apparent on the individual gene level [12]. Therefore, pathway and network-based methods have been developed in an attempt to reveal biological mechanisms underpinning concordant prognosis among distinct gene expression signatures in breast cancer and prostate cancer [14], [15], [16], [17].

Finding common biological themes underlying gene expression signatures lessened earlier worries on the biological validity of the signature genes [18]. Nevertheless, the fact remains that gene signatures determined by supervised data analysis are strongly influenced by the subset of patients used for gene selection, and the membership of a gene in such a signature is not indicative of the importance of that gene in cancer pathology [19]. Because different combinations of genes can be selected to build similarly accurate prediction models [20], an intriguing but unanswered question is whether limiting genomic space to mechanistically important genes can produce accurate prognostic models. A positive answer to this question will lead to better convergence between biological significance and clinical prognosis, which will in turn provide insight into novel targeted therapeutic strategies.

In this work, we studied the biological themes underlying published CRC gene expression signatures. By integrating gene expression signatures and somatic mutation data on a protein-protein interaction network, we show that the CRC recurrence phenotype involves the dysregulation of multiple biological processes, and each signature only captured a few genes in these processes. Based on these observations, we hypothesized that a gene expression signature with mechanistically important genes inferred from network analysis can better represent underlying biology and may lead to prognostic models with improved performance. To this end, we developed Survival Support Vector Machine (SSVM) models using two independent datasets based on such a signature and cross-tested their performance. The results demonstrate that our model can accurately predict CRC recurrence. Moreover, patient stratification based on predicted risk of recurrence provides useful information regarding the adjuvant CTX benefit for CRC patients.

## Methods

### Published CRC Gene Expression Signatures

Through manual literature review on papers published between 2000 and 2010, we identified from seven papers [4], [5], [6], [7], [8], [9], [21] eight gene expression signatures that are able to separate stage II and/or stage III CRC patients into low-risk and high-risk subgroups. The signature in Jorissen et al. [22] was not included because the gene expression datasets used for deriving that signature were used for model development and evaluation in the current study. The eight signatures included a total of 208 genes.

### Genes Mutated in CRC

Using the CanProVar database [23] (http://bioinfo.vanderbilt.edu/canprovar), we retrieved 549 genes with observed somatic mutations in CRC samples.

### Human Protein-protein Interaction Network

Protein interaction data were downloaded and integrated from BioGRID, MINT, HPRD, REACTOME, DIP and MINT in 2010, as previously described [24]. The protein interaction network included 94,066 interactions between 11,521 proteins.

### Oncogenes and Tumor Suppressor Genes

Known oncogenes and tumor suppressor genes were downloaded from CancerGenes [25] and GLAD4U (http://bioinfo.vanderbilt.edu/glad4u). For each tool, we retrieved two gene lists using the query terms oncogene and tumor suppressor, respectively.

### Gene Expression Data Sets

Two gene expression datasets of primary colorectal tumors (GSE17536 [8] and GSE14333 [22]) were downloaded from the Gene Expression Omnibus (GEO) database. Stage I and stage IV samples were excluded from this study. GSE14333 included some of the samples from GSE17536, which were removed from GSE14333 in this study. Clinical and pathological information of the two datasets is shown in Table 1. Both datasets were generated on the Affymetrix U133 plus 2.0 array. cel files for the datasets were normalized using the Robust MultiChip Analysis (RMA) algorithm [26] as implemented in Bioconductor. The datasets were processed separately to ensure their independency. Probe set identifiers (IDs) were mapped to gene symbols based on the mapping provided by the GEO database. Probe sets that mapped to multiple genes were eliminated. When multiple probe sets were mapped to the same gene, the probe set with the largest interquartile range (IQR) was selected owing to its high variation across samples. To make expression level comparable across genes, expression values for each gene were standardized using a Z-score transformation. In this study, each dataset was used as a training-set in turn and developed prognostic models were tested against the other dataset.

**Table 1:** The microarray gene expression datasets used in the study.

	GSE17536 (N = 111)	GSE14333 (N = 67)
**AJCC_STAGE**		
II	55	37
III	56	30
**Recurrence**		
0	80	54
1	31	13
**Adjuvant chemotherapy**		
0	53 (38II+15III)	44 (33II+11III)
1	57 (16II+41III)	23 (4II+19III)
NA	1 (II)	0
**#genes**	19468	19468

AJCC, American Joint Committee on Cancer.

### Network-based Prioritization

**Figure 1:**
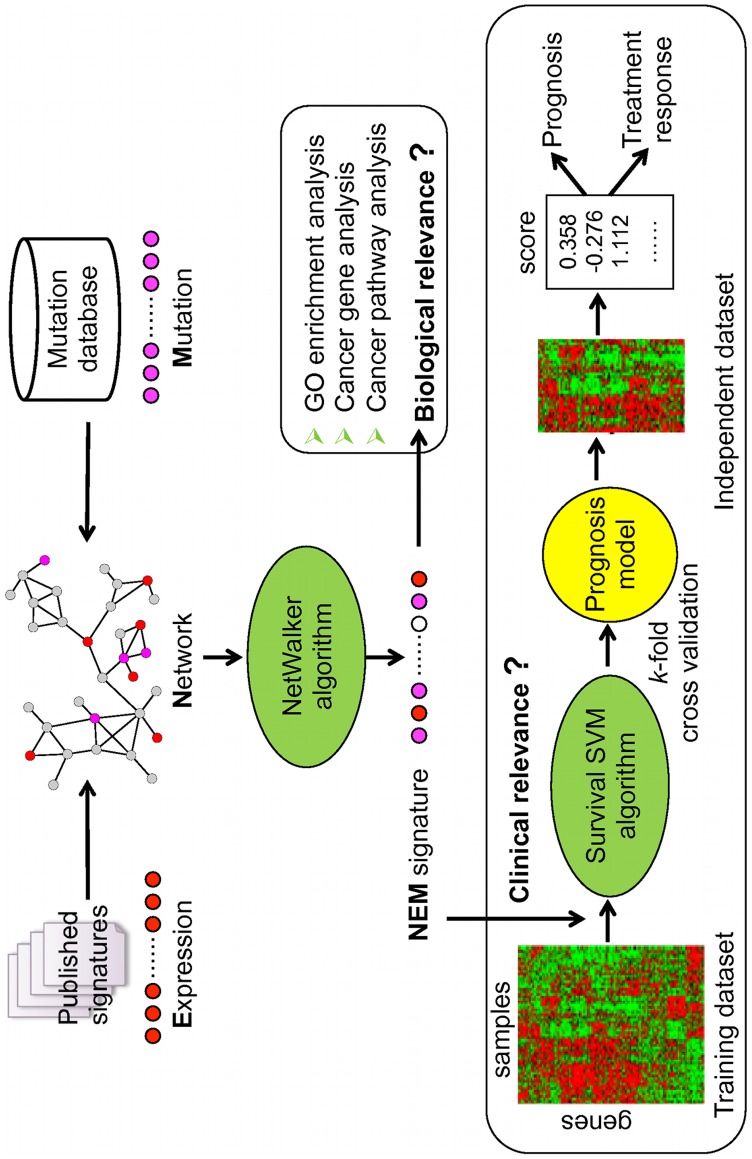
Outline for deriving the network-based signature and validating its biological and clinical relevance. Published gene expression signatures and somatic mutation data were mapped to a protein-protein interaction network. Through integrating information from Mutation, Expression, and Network, a NEM signature was derived using the NetWalker algorithm based on the random walk with restart technique. Biological relevance of the signature was evaluated based on functional information including Gene Ontology, known cancer genes and signaling pathways. Clinical relevance of the signature was evaluated by developing a Survival SVM model based on a gene expression dataset and testing in an independent dataset for its accuracy in prognosis and predicting response to therapy.

We used a modified version of our previously published NetWalker algorithm [24] to integrate expression signatures and publicly available somatic mutation data on a protein-protein interaction network in order to identify genes of potential mechanistic importance to the CRC recurrence phenotype (Figure 1). Netwalker is based on the random walk with restart technique [27]. Given a network and start probabilities for each node representing prior information on their relative importance, the algorithm calculates a final priority score for each node based on the steady state probabilities. Random walk with restart is formally defined as the following equation:
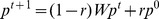
where *r* is the restart probability, *W* is the column-normalized adjacency matrix of the network graph, and *p^t^* is a vector of size equal to the number of nodes in the graph where the *i*-th element holds the probability of being at node *i* at time step *t*.

Although our previous implementation assigns an equal start probability to all seed nodes, this modified version allows different start probabilities for the seed nodes. In this study, we set up the start probabilities for all genes based on their involvement in the gene expression signatures and the mutated gene list. Equal total weight was given to gene expression signature data and mutation data. For gene expression signature data, relatively higher weight was given to genes involved in multiple signatures. For mutation data, relatively higher weight was given to genes with more variants. Start probability for gene *i* (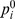) is formally defined as the following equation:
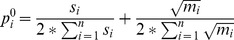
where *s_i_* is the number of CRC gene expression signatures in which gene *i* is a member, *m_i_* is the number of known mutation variants in CRC samples in CanProVar for gene *i*, and *n* is the total number of genes in the protein interaction network.

For the NetWalker algorithm, the restart probability was set to 0.5 and convergence was determined by 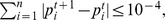 where 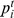 is the probability for gene *i* at the *t*th iteration.

To assess the statistical significance of the scores for each gene, we constructed 1000 sets of randomly permuted start probabilities and generated 1000 sets of random scores. For each gene in the network, a local *p* value was estimated by comparing the real score to random scores from the same gene, and a global *p* value was estimated by comparing the real score to random scores from all genes [24]. Genes with both local and global *p* values less than 0.05 were considered as significant genes. We named the list of significant genes a NEM signature because it integrated information from Network, Expression, and Mutation.

For comparison, we also performed network-based prioritization using start probabilities assigned based only on gene expression signature data or mutation data, respectively, with corresponding significant gene lists named as NE signature or NM signature.

### Gene Ontology Enrichment Analysis

Gene Ontology (GO) enrichment analysis was performed using WebGestalt [28]. The default multiple testing correction method “Benjamini & Hochberg” was used for FDR calculation. To account for the dependent nested GO structure, WebGestalt presents enriched GO categories in a Directed Acyclic Graph (DAG) to facilitate quick identification of the major non-redundant enriched biological themes. We performed a manual investigation of the enriched DAG and reported the most representative terms for each branch.

### Development and Evaluation of SSVM Model

An R implementation of the survsvm available in the survpack package [29], [30] was employed for SSVM model development, and the Gaussian kernel function was used. The implementation of SSVM has two parameters c and σ, where c is the cost of error in the predicted sequence of events and σ is the parameter of the Gaussian kernel. In this study, we let each of these parameters vary among the candidate set {10^−5^, 10^−4^, 10^−3^, 10^−2^, 10^−1^, 10^0^, 10^1^, 10^2^, 10^3^, 10^4^, 10^5^} to form different parameter combinations. Five-fold cross validation was used and repeated five times to identify the optimized parameters according to the C-index value (see below for description). Fully developed SSVM model based on the optimal parameters was then evaluated in the independent dataset where an SSVM-based score was derived for each patient.

### Survival Analysis

The association between the SSVM-based score and real prognosis of the patients was evaluated by the C-index values, Kaplan-Meier survival curves and log-rank test. The C-index is a probability of the concordance between predicted and observed survival, with C-index = 0.5 for random predictions and C-index = 1 for a perfectly discriminating model. Standard Kaplan–Meier survival curves were generated for patient groups formed based on the SSVM scores, and the survival difference between groups was statistically evaluated using the log-rank test.

## Results

### Enrichment Analysis Failed to Reveal Functional Convergence of the Signatures

We investigated 8 CRC gene expression signatures (Table 2). Seven out of the 8 signatures were developed based on the comparison of recurrent and non-recurrent tumors, in which some studies included tumors of all stages while others included only tumors of selected stages. The study by Smith et al. [8] integrated human tumor data with data from CRC mouse cell line models in signature development. The study by Barrier et al. [21] used non-neoplastic mucosa from stage II patients instead of tumors. The *t*-test and its variants were used for signature selection in most of the studies, and different machine learning techniques were employed for the construction of prognostic models. Despite of the technical difference in experimental and computational procedures, all prognostic models were able to separate stage II and/or stage III patients into low-risk and high-risk groups. Several models have been validated on a patient cohort independent of the one used for signature and model development.

**Figure 2:**
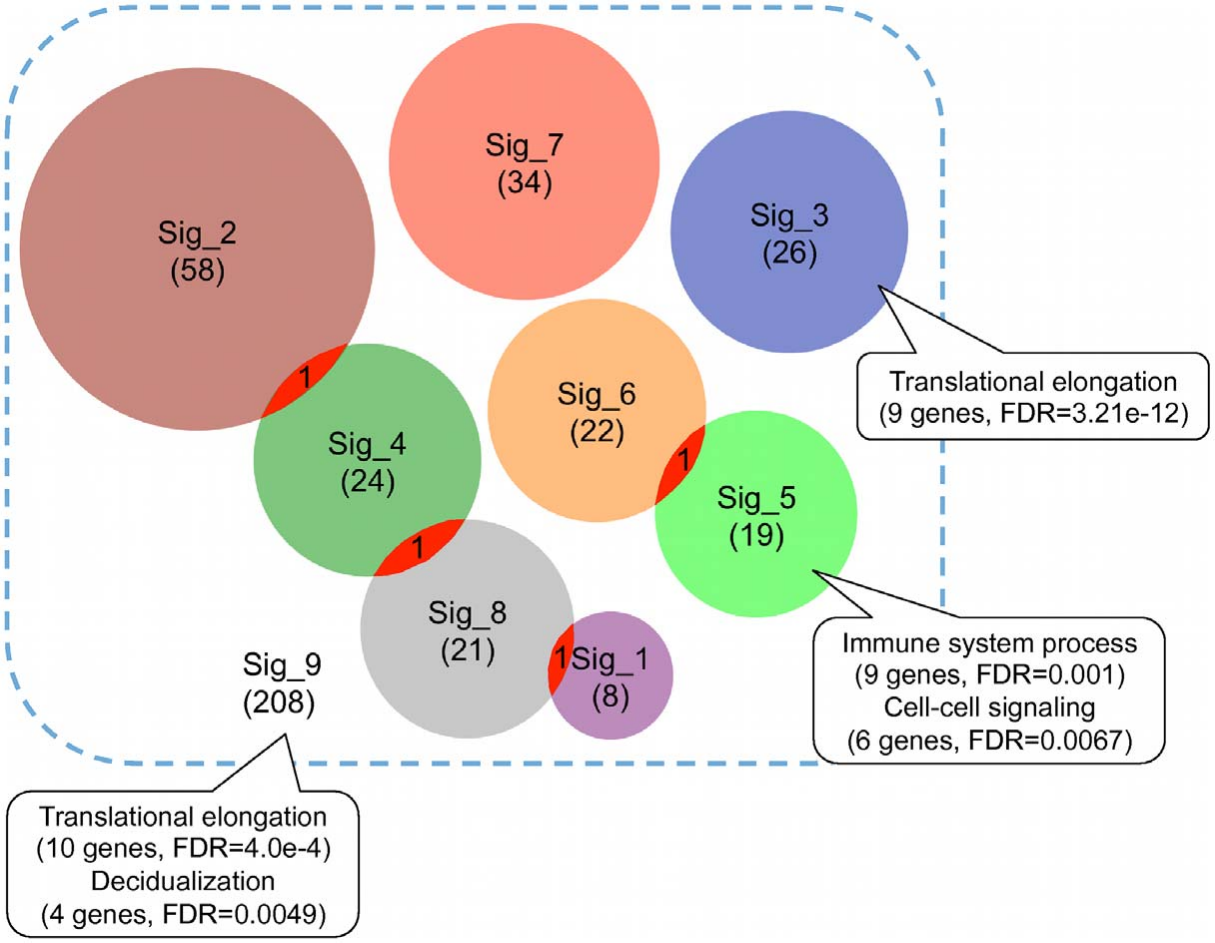
Minimal overlap among published CRC gene expression signatures. Each circle represents one gene expression signature with the number in the parentheses indicating the signature size. The callouts annotate enriched biological processes, numbers of genes involved in the processes, and corresponding False Discovery Rates for the significance of enrichment.

Consistent with previous reports [10], we found minimal overlap among these gene expression signatures at individual gene level (Figure 2). To test whether these signatures converge at common biological processes, we performed Gene Ontology (GO) enrichment analysis for each signature using WebGestalt. Only two signatures showed enriched biological processes at the significance level of False Discovery Rate (FDR) less than 0.01 (Figure 2). Signature_3 was enriched in “translational elongation” (9 genes, FDR = 3.21e-12) and Signature_5 was enriched in “immune system process” (9 genes, FDR = 0.001) and “cell-cell signaling” (6 genes, FDR = 0.0067). Enrichment results from signatures 3 and 5 suggested that different signatures might be associated with different biological mechanisms. Moreover, lack of functional concordance for other signatures indicated that different genes in a signature might represent distinct biological themes and possibly, noise. To further test whether common biological themes could be identified by combining all signatures, we performed enrichment analysis for all 208 genes in the 8 signatures. Enriched biological processes identified included “translational elongation” (10 genes, FDR = 4.0e-4) and “decidualization” (4 genes, FDR = 0.0049). The former was obviously contributed primarily by signature_3. Thus, enrichment analysis failed to reveal functional convergence of the CRC gene expression signatures. Interestingly, although earlier studies reported wide concordance between the biological processes captured by different breast cancer prognostic signatures, a recent study [31] comparing two machine-learning based breast cancer prognostic signatures only found statistically significant concordance in cell proliferation.

**Table 2:** Eight published CRC gene expression signatures.

ID	Array	Sample type	Method	Signaturesize	Signature IDtype	Unique genes	Independent evaluation	Reference
**Sig_1**	Human 19K Oligo array(UMNJ)	Stage II colorectal tumors (6recurrence +10 disease-free)	Genes identified by botht-test and Fisher test	8	Gene Bank ID	8	No	Bandres et al. [4]
**Sig_2**	Affymetrix HG-U133A	Non-neoplastic mucosafrom stage II patients (10recurrence +14 disease-free)	Genes with the largestabsolute t-statistics in t-test	70	Probe set ID	58	No	Barrier et al. [21]
**Sig_3**	Affymetrix HG-U133A	Stage II colorectal tumors (25recurrence +25 disease-free)	Genes with the largestabsolute t statistics in t-test	30	Probe set ID	26	No	Barrier et al. [5]
**Sig_4**	Human 32,488-elementcDNA array (TIGR)	Adenoma + Stage II-IV tumors(48 poor prognosis +30 good prognosis)	Genes consistently selectedby the t-test in LOOCV	43	Gene Bank ID	24	Yes	Eschrich et al. [6]
**Sig_5**	Affymetrix HG-U133A	Stage I-II colorectal tumors(26 recurrence +29 disease-free)	Gene list that generated thebest prediction model (3-NN*classifier model) in LOOCV	19	Gene symbol	19	Yes	Lin et al. [7]
**Sig_6**	Human 30 K Oligoarray (MWG Biotech)	Stage I–IV colorectal tumors(47 recurrence +102 disease-free)	Gene list that generated thebest prediction model (SVMmodel) in LOOCV	22	Gene symbol	22	Yes	Lin et al. [7]
**Sig_7**	Affymetrix MouseGenome 430 2.0;HG-U133 Plus 2.0	Mouse cell line models of CRC(invasive vs non-invasive,3 replicates each); Stage I-IVtumors (19 high risk+36 low risk)	Genes differentially expressedbetween invasive and non-invasive cell lines and showedthe same direction of changein high risk patients	34	Gene symbol	34	Yes	Smith et al. [8]
**Sig_8**	Affymetrix HG-U133A	Stage II colorectal tumors (31recurrence +43 disease-free)	Unsupervised clusteringseparated samples into twogroups; univariate cox modeland t-test were used to selectgenes from each group andthen combined	23	Probe set ID	21	No	Wang et al. [9]

LOOCV: Leave One Out Cross Validation; NN: Nearest Neighbor; SVM: Support Vector Machine; TIGR: The Institute for Genomic Research; UMNJ: University of Medicine of New Jersey.

### Integrative Network Analysis Identified Common Mechanisms Underpinning CRC Recurrence

**Figure 3:**
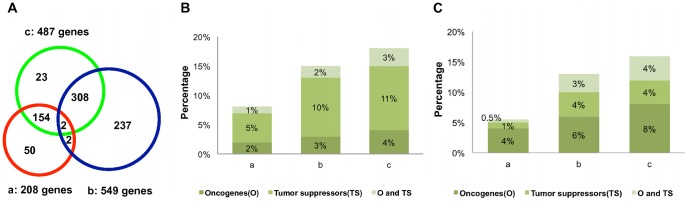
Cancer-relevance of the published gene expression signatures, mutated genes, and the NEM signature. (A) Overlap among the published gene expression signatures (208 genes), mutated genes (549 genes), and the NEM signature (487 genes). (B) The percentage of oncogenes and tumor suppressor genes in the published gene expression signatures (a), mutated genes (b), and the NEM signature (c), as annotated by CancerGenes. (C) The percentage of oncogenes and tumor suppressor genes in the published gene expression signatures (a), mutated genes (b), and the NEM signature (c), as annotated by GLAD4U.

Previous studies suggest that genes known to be associated with the same disease phenotype tend to lie close to each other in a protein-protein interaction network [27], [32]. Furthermore, Chen et al. [16] showed that cancer signature genes are more likely to be close to known oncogenes and tumor suppressors in a protein-protein interaction network. Therefore, we used a network-based approach to integrate these signatures on the protein-protein interaction network in an attempt to identify genes of potential mechanistic importance to the CRC recurrence phenotype. In addition to gene expression alteration, somatic mutations in mechanistically important genes may also lead to the same phenotype. Therefore, we further collected 549 genes with somatic mutations in CRC from the CanProVar database [23] to enhance the network analysis using the NetWalker algorithm [24]. Both signature gene lists and the mutated gene list included mechanistically important genes (e.g. driver mutations and effectors) and other genes (e.g. passenger mutations and epiphenomena). Moreover, some mechanistically important genes might be missing in these lists. The NetWalker algorithm infers genes of potential mechanistic importance based on the assumption that these genes are likely to form tightly connected clusters while others tend to be randomly distributed on the network. Using the signature genes and the mutated genes as “seeds”, the algorithm calculated a score for each gene in the network based on its overall proximity to all seed genes, where the proximity is measured by the random walk similarity [27]. To assess the statistical significance of the scores, we constructed 1000 sets of random seeds and generated 1000 sets of random scores. For each gene, we estimated a local *p* value based on all random scores of the same gene and a global *p* value based on random scores for all genes. A significant global *p* value indicates the overall significance of the gene with regard to the input seeds, while a significant local *p* value ensures that the significance is not simply due to network topology [24]. A total of 487 genes with both local and global *p* values less than 0.05 were considered as significant genes, including 464 from the original lists and 23 added by the algorithm (Figure 3A). We named the list of 487 genes the NEM signature because it integrated information from Network, Expression, and Mutation. The list included well-known CRC-related genes, including APC, CTNNB1, KRAS, TP53, BRAF, among others. It also included genes with unknown but potential importance in CRC recurrence. A complete list of the NEM signature genes and their *p* values are available in Table S1. To test the robustness of the method with regard to different input gene expression signature lists, we removed each expression signature from the seeds, one at a time, and generated 8 NEM-7 signatures (thus named because they used only 7 out of the 8 available gene expression signatures). These experiments altered the total number of input expression signature genes from 4% (when signature_1 was removed) to 28% (when signature_2 was removed). The Dice’s coefficient between the NEM-7 signatures and the original NEM signature ranged from 0.88 to 0.96, with a mean of 0.93, suggesting high robustness of the method.

GO enrichment analysis of the NEM signature identified four major biological processes with significant enrichment (Table 3), including “signal transduction” (186 genes, FDR = 7.07e-11), “cell proliferation” (71 genes, FDR = 3.03e-8), “programmed cell death” (75 genes, FDR = 1.83e-9), and “developmental process” (158 genes, FDR = 3.98e-9). Although these processes are broad and not necessarily cancer-specific, they are consistent with the hallmarks of cancer [33]. Except for Signature_1, all other expression signatures included a small number of genes in some or all of these biological processes (Table 3). Moreover, all these biological processes were significantly enriched in all of the NEM-7 signatures.

**Table 3:** Number of genes associated with the enriched GO terms for the NEM signature.

Signature ID	Signature size	Signal transduction	Cell proliferation	Programmed cell death	Developmental process
**NEM signature**	487	186	71	75	158
**Sig_1**	8	0	0	0	0
**Sig_2**	58	9	6	3	9
**Sig_3**	26	6	2	2	4
**Sig_4**	24	7	1	2	3
**Sig_5**	19	7	5	3	6
**Sig_6**	22	3	0	0	1
**Sig_7**	34	5	5	3	11
**Sig_8**	21	2	1	0	3

Next, we calculated the ratios of known oncogenes and tumor suppressor genes in the union of published gene expression signatures, the somatic mutation gene list, and the NEM signature, based on annotations from two different resources, CancerGenes and GLAD4U. Because many of the known oncogenes and tumor suppressor genes are identified based on somatic mutation, it was not surprising that the somatic mutation gene list had a higher percentage of these genes than the gene expression signatures. However, it was interesting to see that the NEM signature had the highest percentage of known oncogenes and tumor suppressor genes (Figure 3, B–C). To better understand the involvement of the NEM signature genes in cancer-specific pathways, we mapped them to the cancer pathway map curated by KEGG. As shown in Figure S1, the gene list mapped to nearly all of the cancer-related pathways, with a clear enrichment in the Wnt signaling pathway, the TGF-beta signaling pathway, and the ErbB signaling pathway, the most important pathways that are deregulated in CRC [34]. In summary, the NEM signature showed significant enrichment in four biological processes closely related to cancer pathophysiology and provided good coverage of known oncogenes, tumor suppressors, and CRC-related signaling pathways, thus demonstrating a high relevance to CRC biology.

### The NEM Signature-based Prognostic Models Effectively Predicted CRC Recurrence

To test whether the NEM signature with genes centered on functionally important networks can predict CRC recurrence, we developed prognostic models using these genes as features and evaluated performance of the models in independent patient cohorts.

**Figure 4:**
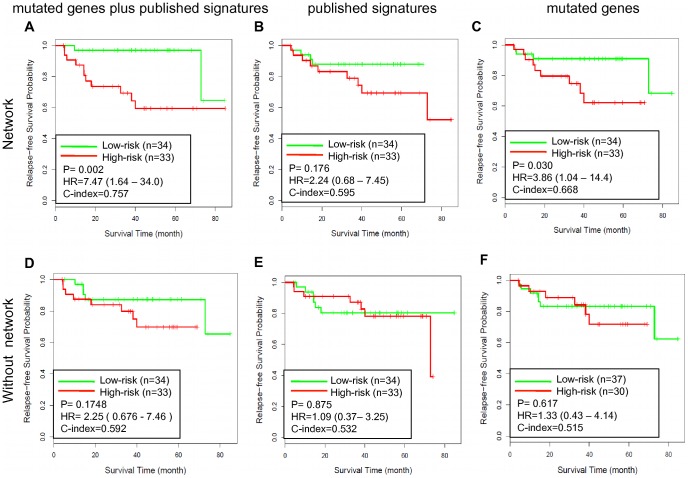
Testing the GSE17536-derived SSVM prognostic models on GSE14333. Kaplan-Meier survival curves for patient subgroups identified in GSE14333 using models developed based on GSE17536 with different gene sets. (A) The NEM signature based on network analysis with the seed nodes including 208 genes in published signatures and 549 mutated genes, N = 487; (B) The NE signature based on network analysis with the seed nodes including 208 genes in published signatures, N = 546; (C) The NM signature genes based on network analysis with the seed nodes including 549 mutated genes, N = 435; (D) the union of 208 genes in published signatures and 549 mutated genes, N = 753; (E) 208 genes in published signatures, N = 208; (F) 549 mutated genes from CanProVar, N = 549.

First, we trained a SSVM prognostic model using gene expression dataset GSE17536 and tested its performance on an independent data set GSE14333. Among the 487 genes in the NEM signature, only the 467 genes in the dataset were used to train the model. Five-fold cross validation was used and repeated 5 times to optimize the parameters for the SSVM algorithm, and a full model based on the complete dataset was developed using the optimal parameters. For testing in GSE14333, SSVM scores were calculated for individual samples, with a higher score indicating higher risk and shorter survival time. The calculated SSVM scores and the real survival data showed 75.7% concordance (C-index = 0.757). Based on the SSVM scores, the patients were separated into two groups, a “low-risk” group with below-median scores and a “high-risk” group with above-median scores. As shown in Figure 4A, the high-risk group had significantly worse relapse-free survival (hazard ratio [HR], 7.47; 95% confidence interval [CI], 1.64–34.0; P = 0.002) than the low-risk group. The relapse free survival at 3 years was 96.9% for the low-risk group compared with 69.3% for the high-risk group.

**Figure 5:**
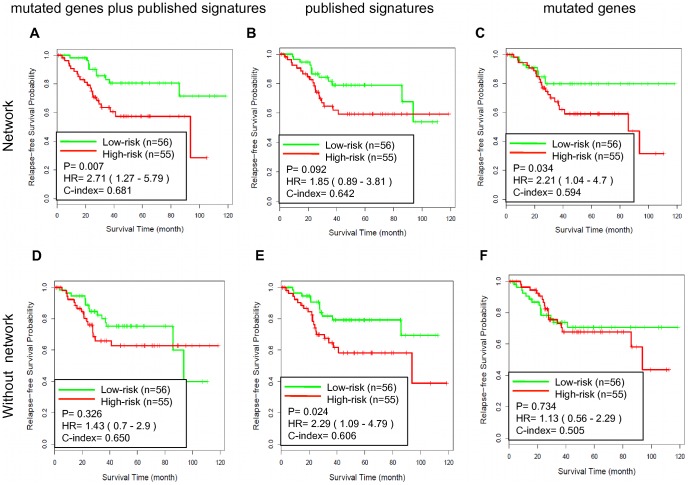
Testing the GSE14333-derived SSVM prognostic models on GSE17536. Kaplan-Meier survival curves for patient subgroups identified in GSE17536 using models developed based on GSE14333 with different gene sets. (A) The NEM signature based on network analysis with the seed nodes including 208 genes in published signatures and 549 mutated genes, N = 487; (B) The NE signature based on network analysis with the seed nodes including 208 genes in published signatures, N = 546; (C) The NM signature genes based on network analysis with the seed nodes including 549 mutated genes, N = 435; (D) the union of 208 genes in published signatures and 549 mutated genes, N = 753; (E) 208 genes in published signatures, N = 208; (F) 549 mutated genes from CanProVar, N = 549.

**Table 4:** Univariate and multivariate Cox proportional hazard regression analyses of relapse-free survival in GSE14333.

	Univariate		Multivariate	
	*p* value	HR (95% CI)	*p* value	HR (95% CI)
Age	0.552	1.02 (0.97–1.07)	0.693	1.00 (0.96–1.06)
Gender (M or F)	0.951	1.04 (0.34–3.18)	0.792	0.85 (0.27–2.74)
AJCC STAGE (II, III)	0.012	4.52 (1.24–16.46)	0.012	5.51 (1.46–20.85)
NEM signature score	0.002	7.47 (1.64–34.0)	0.007	9.40 (1.86–47.63)

AJCC, American Joint Committee on Cancer; F, female; M, male; NEM signature score was based on SSVM models developed in GSE17536.

A recent study suggests that most random gene expression signatures are significantly associated with breast cancer outcome [35]. Therefore, we repeated our analysis using 10 sets of randomly selected 487 genes. When the models trained on GSE17536 were tested on GSE14333, they got a median C-index of 0.546 and a median P value of 0.568. Thus, random gene signatures do not seem to work in CRC prognosis.

One consideration is that 487 genes might be too many for practical clinical implementation. Therefore, we tried different cutoff values in the network-based prioritization process to alter the number of selected genes. Using different *p* value cutoffs including 0.005, 0.01, and 0.1, we identified 45, 105 and 810 genes, respectively. Using parameters selected based on cross-validation results, three SSVM models were developed on GSE17536 and tested on GSE14333 respectively. As shown in Figure S2, the performance of the 810 gene model was comparable to that of the 487 gene model, while the 105 and 45 gene models showed little prediction power. Therefore, further reducing the genomic space seems problematic, possibly due to the underlying complexity of CRC.

**Figure 6:**
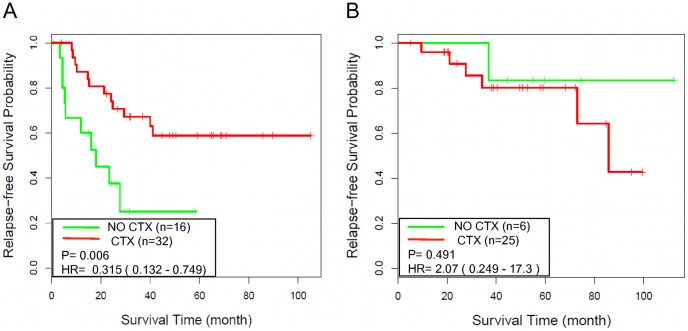
NEM signature score-based risk stratification informs response to adjuvant chemotherapy (CTX). (A) Kaplan-Meier survival curves for high-risk patients in GSE17536 and GSE14333, with (CTX) and without (NO CTX) adjuvant CTX; (B) Kaplan-Meier survival curves for low-risk patients in GSE17536 and GSE14333, with and without adjuvant CTX.

Because the NEM signature integrated information from mutations, gene expression signatures, and the protein-protein interaction network, we tried to dissect their individual contribution to the observed performance. Network signatures derived using the same network prioritization method but based on either the gene expression signatures alone (NE signature with 546 genes, Figure 4B) or the mutated genes alone (NM signature with 435 genes, Figure 4C) did not result in comparable performance as that from the NEM signature (Figure 4A). Specifically, the C-index for the NEM signature-based model was 27% higher than that for the NE signature-based model and 13% higher than that for the NM signature based model. On the other hand, all three models derived from network signatures (Figure 4A–C) performed better than their counterparts without network-based prioritization (Figure 4D–F). For example, the C-index for the NEM signature-based model was 28% higher than that for the model based on the union of all gene signatures and mutated genes. These results suggest that network-based prioritization facilitates achieving the observed performance, and both expression signatures and mutated genes provide complementary information during the network-based prioritization. Thus, information from all three sources plays important roles in deriving the biologically and clinically relevant signature.

The network analysis only added 23 genes to the NEM signature, suggesting that the main effect of the analysis was to filter out noisy or functionally irrelevant genes. However, it is of particular interest whether the 23 added genes contributed to the good performance of the NEM signature. To answer this question, we removed the 23 genes from the NEM signature and repeated the analysis. The new model based on the remaining genes showed deteriorated performance (P = 0.121; HR = 2.47, 95% CI = 0.76–8.02; C-index = 0.632). Thus, the 23 genes added by the network analysis indeed contributed to the prognostic performance of the NEM signature.

To further evaluate the effectiveness of the NEM signature, we reversed the training and testing datasets by training on gene expression dataset GSE14333 and testing on gene expression dataset GSE17536. Analogous results were obtained as shown in Figure 5. Scores derived from the NEM signature-based model and the real survival data showed 68.1% concordance (C-index = 0.681) and separated the patients into two groups with significantly different relapse-free survival (hazard ratio [HR], 2.71; 95% confidence interval [CI], 1.27–5.79; P = 0.007). The relapse free survival at 3 years was 82.9% for the low-risk group compared with 63.5% for the high-risk group. All three models derived from network signatures (Figure 5A–C) attained a better C-index than their counterparts without network-based prioritization (Figure 5D–F). Two out of the three also resulted in a better P value. The NEM signature-based model performed the best among all models. These results confirmed that the NEM signature-based prognostic models could effectively predict CRC recurrence.

**Figure 7:**
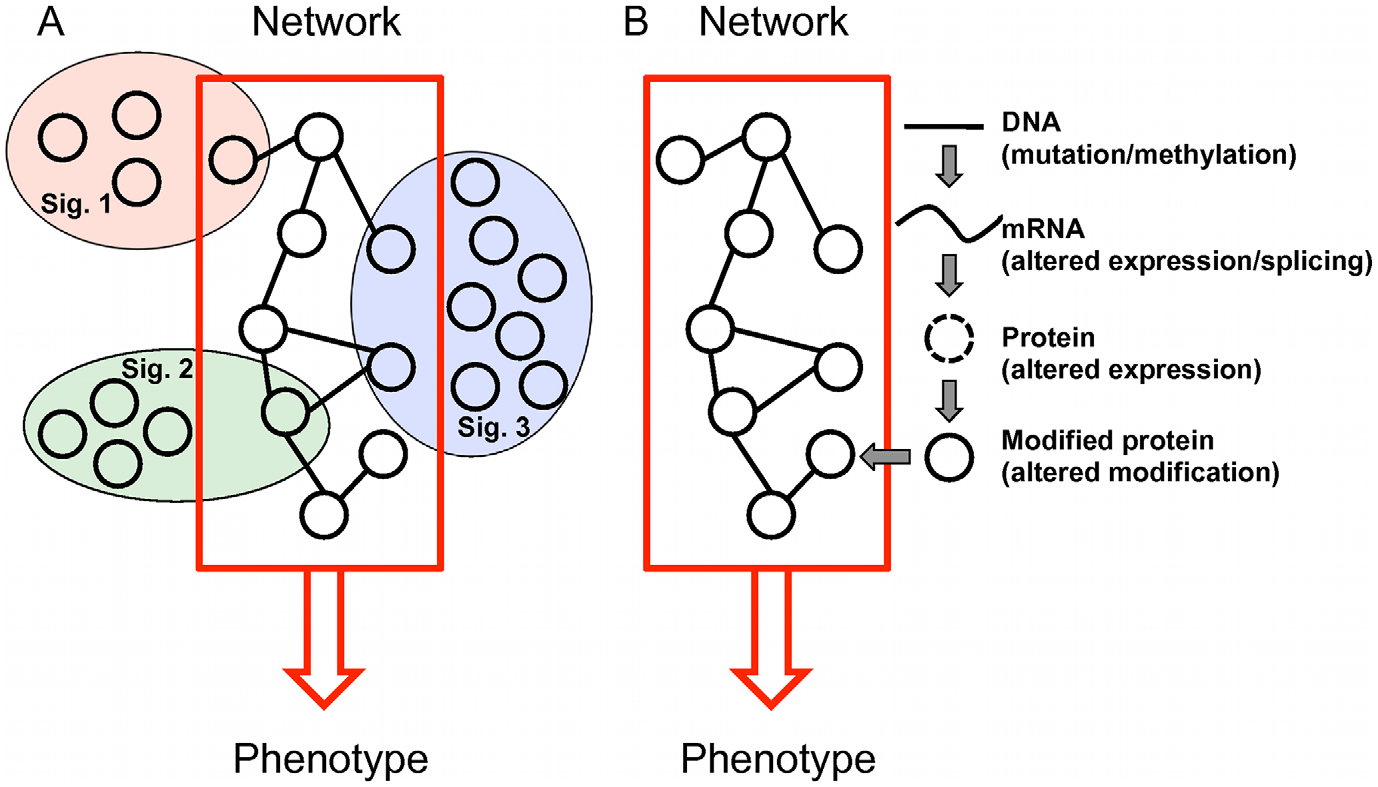
Protein interaction network as a model for data integration. (A) Individual gene expression signatures only include a small number of components related to one or a few of critical mechanisms, thus demonstrate minimal gene-level concordance. (B) Molecular alterations at different levels eventually exert their effects through altered protein and network activity.

### Prognostic Value of the NEM Signature Score Compared to Clinical Variables

Univariate and multivariate Cox proportional hazards regression analyses were applied to GSE14333 to evaluate the prognostic value of the NEM signature score in combination with clinical variables including patient age at diagnosis, gender and AJCC stage, where the NEM signature score for the samples were derived from the NEM signature-based model developed on GSE17536. In the univariate analysis, the NEM signature score and AJCC stage were significantly associated with relapse-free survival (*p* = 0.002 and *p* = 0.012, respectively). In the multivariate analysis, the NEM signature score and AJCC stage still maintained the significance (*p* = 0.007 and *p* = 0.012, respectively) (Table 4). According to the log-rank *p* values, the NEM signature score was more significantly associated with relapse-free survival than AJCC stage. Thus, the NEM signature score contributed more information about recurrence than standard clinical and pathologic covariates.

### NEM Signature Score-based Risk Stratification Provided Insight into the Response to Adjuvant CTX

Finally, we tested whether the NEM signature score-based risk stratification could provide insight into the benefit from adjuvant CTX. This analysis was limited to stage III colon cancer patients with adjuvant CTX information. Stage II patients were excluded because very few of them received adjuvant CTX. Rectal cancer patients were excluded to avoid potential confounding effects of treatment differences. According to the NEM signature-based prognostic models, 31 patients (10 from GSE14333 and 21 from GSE17536) were assigned to the low-risk group and 48 patients (13 from GSE14333 and 35 from GSE17536) were assigned to the high-risk group. Interestingly, 81% (25 out of 31) of patients in the low-risk group received adjuvant CTX compared to 67% (32 out of 48) in the high-risk group. As shown in Figure 6A, adjuvant CTX was significantly associated with prolonged survival of patients in the high-risk group (hazard ratio [HR], 0.32; 95% confidence interval [CI], 0.13–0.75; P = 0.006), with a 67% relapse free survival rate at 3 years for patients received CTX compared to 25% for patients did not receive adjuvant CTX. In contrast, adjuvant CTX was not beneficial to patients in the low-risk group (hazard ratio [HR], 2.07; 95% confidence interval [CI], 0.25–17.3; P = 0.491), with a 80% relapse free survival rate at 3 years for patients who received adjuvant CTX as compared to 83% for patients did not receive CTX (Figure 6B). Although this result needs to be further evaluated in additional patient cohorts with larger sample size, we concluded based on available data that the NEM signature score-based risk stratification could provide useful information on potential benefit from adjuvant CTX.

## Discussion

A major conclusion from this study is that accurate prognostic models can be developed using mechanistically important genes inferred from our network analysis. Although the NEM signature was not directly selected for optimizing prediction performance, prognostic models based on the signature effectively predicted CRC recurrence and provided useful insight into patient response to adjuvant CTX.

The network-based approach used for the NEM signature development has two distinct advantages. First, it provides a knowledge-driven method for reducing feature space dimensionality. Owing to the large number of genes and small sample size, reducing the gene dimensionality is a necessary step in microarray based classification studies. Existing methods usually select genes to optimize prediction performance in the training dataset (e.g. through cross-validation); in contrast, our approach selects genes based on their functional importance inferred from network analysis. Therefore, gene selection in existing methods depends on the training data, whereas in our approach, it is knowledge-driven and independent of the training data. Although functionally important genes do not necessarily show expression change in a specific tumor sample, expression alteration among these genes are most likely to cause downstream phenotype changes. Secondly, the network-based signature provides biological insight about the disease. The NEM signature showed significant enrichment in four important biological processes related to cancer pathology. It also provided good coverage of known oncogenes, tumor suppressors, and CRC related signaling pathways. Although a biological explanation is not strictly necessary for a successful prediction model [36], such information may help identify potential new targets for drug development in follow-up studies.

Our analysis also offers biological explanations for the small overlap of the published CRC gene expression signatures and the lack of biological interpretation for these signatures (Figure 7A). Enrichment analysis of the NEM signature suggests that the CRC recurrence phenotype is the result of dysregulation of multiple important biological processes. Individual signatures only include a small number of components related to one or a few of these mechanisms, thus demonstrate minimal gene-level concordance. Moreover, although each signature carries information on gene expression changes in the important mechanisms, these critical changes have been mixed with multitude of secondary or adaptive changes, thus prevent the identification of the common biological themes in GO enrichment analysis. Because gene expression alterations exert their effects primarily by changing the levels and activity of proteins and their participating networks, network-based analysis provides an effective approach to distinguish genes of potential mechanistic importance from gene showing secondary or adaptive changes. In addition to gene expression change, other alterations at DNA, mRNA, and protein levels can all lead to protein function alteration and, in turn, network dysregulation (Figure 7B). Therefore, the network-based prioritization provides a common framework that can be used to integrate genomic, transcriptomic, and proteomic data sets relevant to a common pre-defined cancer phenotype in order to highlight biological mechanisms underlying the phenotype of interest and pinpoint important genes for gene signature development. Such a signature can be used for the development of gene expression-based prognostic models, as described in this study. Moreover, with the availability of matched expression and mutation data from the same patients, one can use such a signature to build more powerful models based on both gene expression and mutation status of the genes.

Notwithstanding its great potential, the network-based signature development approach is limited by the quality of currently available data. Although the performance of the NetWalker algorithm has been demonstrated to be robust against false positive and false negative interactions in the protein-protein interaction network [24], we believe that the growing effort in protein-protein interaction network studies and concomitant increase of network coverage and quality should improve the prioritization result. The somatic mutation data used in the study was downloaded from our previously developed CanProVar database that integrated cancer related mutations from various resources [23]. We found that only approximately 25% of the mutations were supported by two or more data sources, suggesting a high ratio of false positives and false negatives of the dataset. Ongoing large-scale cancer genome projects, such as the Cancer Genome Project (CGP) of the Sanger Institute and The Cancer Genome Atlas (TCGA) project of the NCI and NHGRI could rapidly increase the coverage and quality of the somatic mutation data. Almost all of the gene signatures used in this study were developed based on the comparison of microarray data from highly aggressive and less aggressive CRC tumors. Recently, new CRC signatures have been published based on the same approach [22], [37], unsupervised analysis of CRC subtypes [38], or model system based mechanistic analysis [39]. It would be useful to add these signatures to our network-based prioritization to refine our NEM signature.

Because of high heterogeneity, stage II and III CRC patients can significantly benefit from an accurate risk evaluation model. Genomic markers currently used to support decision-making in CRC treatment include the microsatellite instability (MSI) that is associated with lack of efficacy of adjuvant CTX, and KRAS mutations that are predictive of lack of effectiveness of epidermal growth factor receptor (EGFR) inhibitors. Both of these are only useful as negative markers and cannot predict the subset of patients who will benefit from adjuvant CTX [40], [41]. Our model provides insight into both negative and positive responses to adjuvant CTX and therefore could support clinical decision making after further evaluation in patient cohorts with larger sample size.

## Supporting Information

Figure S1 **Pathways in cancer (from KEGG) with genes in the MEN signature highlighted in red.**
(PDF)

Figure S2 **Testing the GSE17536-derived SSVM prognosis models on GSE4333.** Kaplan-Meier survival curves for patient subgroups identified in GSE14333 using models developed based on GSE17536 with genes selected according to different significance cutoff values. (A) 0.005; (B) 0.01; (C) 0.1.
(PDF)

Table S1 **NEM signature genes and their** ***p*** **values.**
(XLSX)
