# Supplementary figures and images for: A Network-Based Gene Expression Signature Informs Prognosis and Treatment for Colorectal Cancer Patients
Source: PLoS One. 2012 Jul 23;7(7):e41292. doi: 10.1371/journal.pone.0041292 (PMC3402487; doi:10.1371/journal.pone.0041292)

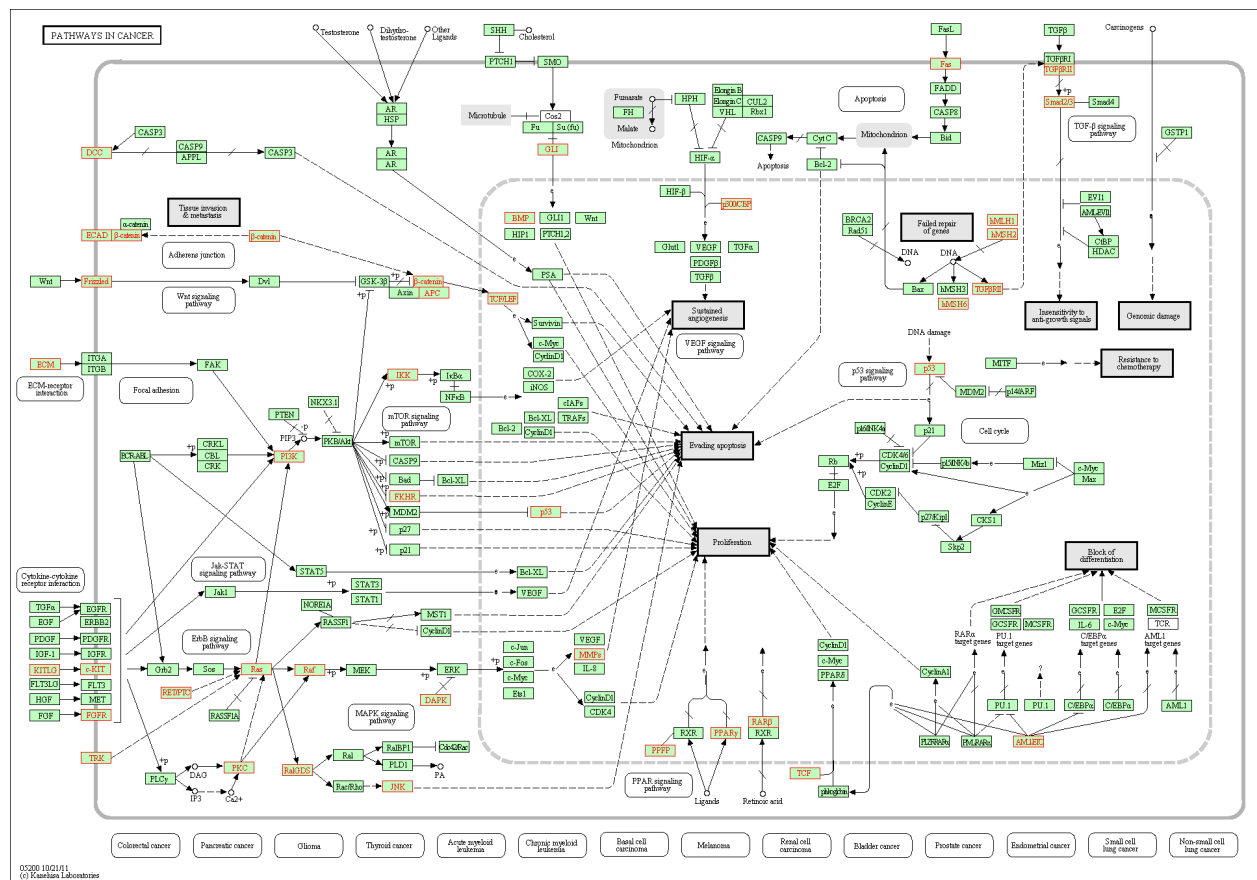

**Figure S1. Pathways in cancer (from KEGG) with genes in the MEN signature highlighted in red.**

Supplement: Figure S1 — Pathways in cancer (from KEGG) with genes in the MEN signature highlighted in red. (PDF) [file pone.0041292.s001.pdf]
