# Supplementary material for: A Network-Based Gene Expression Signature Informs Prognosis and Treatment for Colorectal Cancer Patients
Source: PLoS One. 2012 Jul 23;7(7):e41292. doi: 10.1371/journal.pone.0041292 (PMC3402487; doi:10.1371/journal.pone.0041292)
Supplement: Figure S2 — Testing the GSE17536-derived SSVM prognosis models on GSE4333. Kaplan-Meier survival curves for patient subgroups identified in GSE14333 using models developed based on GSE17536 with genes selected according to different significance cutoff values. (A) 0.005; (B) 0.01; (C) 0.1. (PDF) [file pone.0041292.s002.pdf]

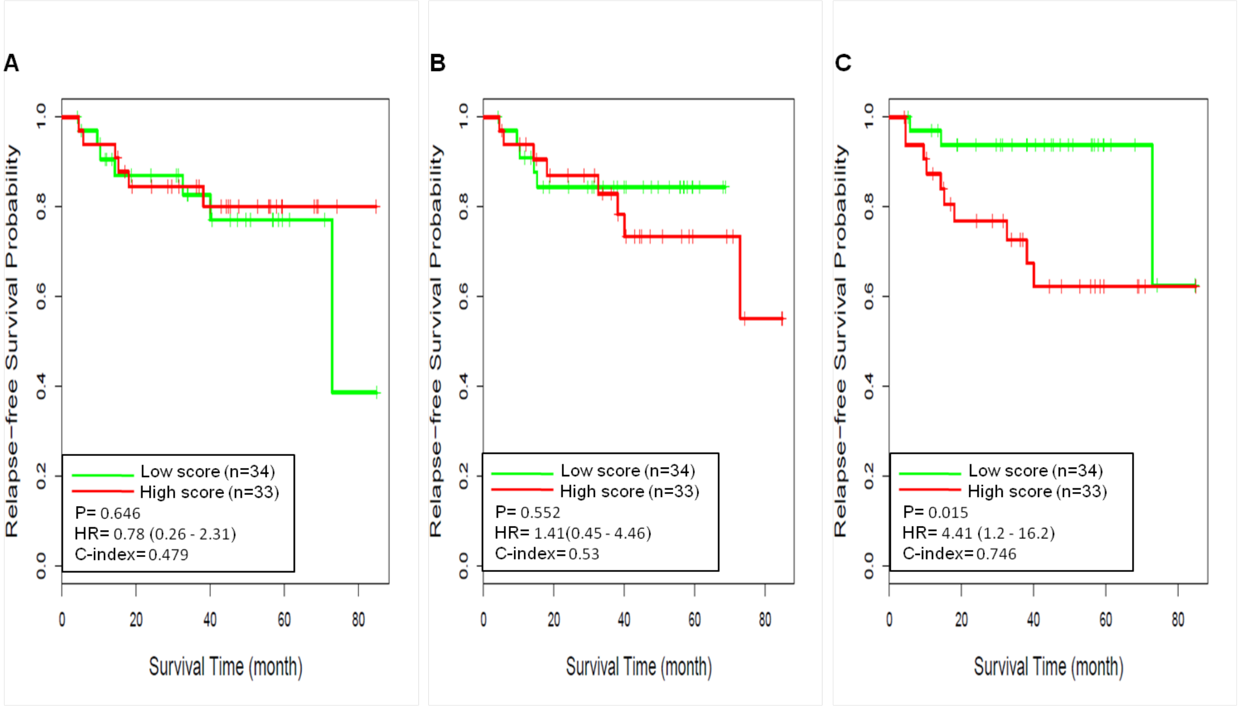

Figure S2. Testing the GSE17536-derived SSVM prognosis models on GSE4333. Kaplan-Meier survival curves for patient subgroups identified in GSE14333 using models developed based on GSE17536 with genes selected according to different significance cutoff values. (A) 0.005; (B) 0.01; (C) 0.1.
